# How much Sistan was successful in tuberculosis control?

**DOI:** 10.1186/2047-2994-4-S1-P101

**Published:** 2015-06-16

**Authors:** A Sargazi, Z Sepehri, A Sargazi, PK Nadakkavukaran Jim, Z Kiani

**Affiliations:** 1 Medical faculty, Zabol University of Medical Sciences, Zabol, Islamic Republic Of Iran; 2 Research department, Zabol University of Medical Sciences, Zabol, Islamic Republic Of Iran; 3 Civil faculty, Sistan and Baloochestan University, Zahedan, Islamic Republic Of Iran; 4 Oracle e-Business Department, Tech Mahindra, Linlithgow, UK; 5 Medical faculty, Kerman Medical University, Kerman, Islamic Republic Of Iran

## Introduction

Tuberculosis (TB) is known as an infectious disease with high burden from a long time.

## Objectives

Various strategies are implemented to control its spread in the world. Tuberculosis is controlled in Sistan by implementing Tuberculosis Control Program in Sistan (TCPS) and this study is to evaluate TCPS and Tuberculosis worker's role in it.

## Methods

For this purpose we collected data with the methods of questionnaires, checklists, interviews, documentation review and observation. Using these methods together with data collection will boost the advantages of each single method. Effectiveness and efficiency considered as the priorities of TCPS evaluation. Data analyzed by using SPSS software.

## Results

Current study showed in the first year of implementation they could run about 80% of the program quantitatively. The TB incidence was 94 and 107 per 100,000 of population respectively in 2011 and 2012 one year before and after TCPS implementation. The TCPS center reported 92.6% and 88.4% of TB new cases in 2011 and 2012 respectively showed from the each dollar we spend in TCPS implementation, about 2 dollar was saved. The TCPS implemented 25% of WHO recommended strategies.

## Conclusion

The total incidence increased one year after TCPS but the TCPS center and its workers' role in case reporting decreased. It could be related to more awareness in society or total TB growth and more preference and trust on private clinics. However there is no significant change in TB situation or patient destiny after TCPS implementation. The TCPS fail could be related to TB contact screen miss, its distance from World Health Organization procedures and health workers' role.

## Disclosure of interest

None declared.

## References

[B1] Lönnroth K Raviglione M Global epidemiology of tuberculosis: prospects for control. In Seminars in respiratory and critical care Medicine 2008 29 5 Thieme Medical Publishers 481 491 10.1055/s-0028-1085700 18810682

[B2] Atun R Lennox-Chhugani N Drobniewski F Samyshkin Y Coker R A framework and toolkit for capturing the communicable disease programmes within health systems tuberculosis control as an illustrative example The European Journal of Public Health 2004 14 3 267 273 10.1093/eurpub/14.3.267 15369032

